# Anti-Tumor Effect of Non-Thermal Atmospheric Pressure Plasma-Activated Medium on Synovial Sarcoma: An In Vitro and In Vivo Study

**DOI:** 10.3390/biomedicines13030534

**Published:** 2025-02-20

**Authors:** Hana Yao, Hiromitsu Toyoda, Naoki Takada, Naoto Oebisu, Kumi Orita, Yoshitaka Ban, Kosuke Saito, Katsumasa Nakazawa, Yuto Kobayashi, Hiroshi Taniwaki, Chinatsu Ohira, Jun-Seok Oh, Tatsuru Shirafuji, Hidetomi Terai, Hiroaki Nakamura

**Affiliations:** 1 Department of Orthopaedic Surgery, Graduate School of Medicine, Osaka Metropolitan University, Osaka 545-8585, Japann_takada17@hotmail.com (N.T.);; 2 Department of Orthopaedic Surgery, Osaka City Juso Hospital, Osaka 532-0034, Japan; 3 Department of Physics and Electronics, Graduate School of Engineering, Osaka Metropolitan University, Osaka 558-8585, Japan; jsoh@omu.ac.jp (J.-S.O.);

**Keywords:** anti-tumor effect, cell apoptosis, intracellular reactive oxygen species (ROS), non-thermal atmospheric pressure plasma, plasma-activated medium (PAM), synovial sarcoma

## Abstract

**Background/Objective**: Anti-tumor effects of plasma-activated medium (PAM) were demonstrated using various malignant tumors. However, the anti-tumor effect of PAM on synovial sarcoma remains unclear. Therefore, we aimed to investigate the anti-tumor effects of PAM on synovial sarcoma and its underlying mechanisms, focusing on the quantitative analyses of both intracellular reactive oxygen species (ROS) and cell apoptosis. **Methods**: The human synovial sarcoma cell line HS-SY-II was used to investigate the cell viability after PAM treatment. We investigated the anti-tumor effects and side effects of local PAM injection in a synovial sarcoma xenograft murine model. Moreover, we observed PAM-induced intracellular ROS accumulation and cell apoptosis and assessed the involvement of intracellular ROS in the anti-tumor effects of PAM using an intracellular ROS scavenger. **Results**: PAM significantly decreased the viability of synovial sarcoma cells compared with untreated Dulbecco’s modified Eagle medium. Local PAM injection into a synovial sarcoma xenograft murine model significantly suppressed tumor growth, including tumor volume (*p* < 0.001) and weight (*p* = 0.031), without side effects. Regarding anti-tumor mechanisms, PAM induced significant cell apoptosis and intracellular ROS accumulation (*p* < 0.001). The intracellular ROS scavenger significantly inhibited the anti-tumor effect of PAM (*p* < 0.001). **Conclusions**: We confirmed the anti-tumor effects of PAM on synovial sarcoma in vitro and in vivo, as well as the absence of side effects. The underlying mechanism was suggested to involve cell apoptosis induced by intracellular ROS accumulation. Considering the various clinical issues associated with the existing treatments of synovial sarcoma, PAM is a promising new option.

## 1. Introduction

Synovial sarcoma is one of the uncertain differentiated soft tissue sarcomas [1], and accounts for 5–10% of soft tissue sarcomas [2]. Surgical resection with negative margins is the main treatment for synovial sarcoma [3]; however, it can often be invasive and difficult because most synovial sarcomas occur in deep soft tissues of the extremities [1]. Chemotherapy and radiotherapy have limited efficacy in synovial sarcomas [3,4,5,6], and, most importantly, both treatments frequently induce many side effects [7,8,9]. Furthermore, despite the development of multimodal treatment using those treatments, the prognosis of synovial sarcoma remains unsatisfying in adults; 5-year and 10-year survival rates are 53–75.5% and 52–58%, respectively [10,11,12,13].

Recently, non-thermal atmospheric pressure plasma (hereinafter called “plasma”) has been extensively studied for various medical purposes [14,15,16,17,18,19,20,21,22]. Over the last two decades, plasma has attracted the attention of oncologists, and the anti-tumor effects of direct plasma irradiation on various malignant tumors have been reported in vitro [23,24,25,26] and in vivo [27,28]. However, since Tanaka et al. introduced plasma-activated medium (PAM), a cell culture medium irradiated with plasma, which showed anti-tumor effects in glioblastoma cells [29], PAM has become an important research subject for oncologists.

The anti-tumor effect of plasma can be executed in two different ways: direct plasma irradiation of the tumor or indirect injection of a plasma-activated solution in the tumor. In the case of direct plasma treatment, the plasma first reacts with ambient air when it emerges into the atmosphere. This generates various ionic species and short-lived reactive species, including atomic oxygen and atomic nitrogen, hydroxyl radicals, hydrogen radicals, and nitric oxide in the gas phase [30,31]. These short-lived reactive species can be delivered into the tumor. It depends on the effective lifetime of the short-lived reactive species; it can be obtained in the secondary reactions with the gas and liquid interface. As a result, hydrogen peroxide (H_2_O_2_), nitrate (NO_3_^−^), and nitrite (NO_2_^−^) are generated in the tissue, finally reaching the cells [32,33,34]. Unlike direct plasma irradiation, PAM can be easily applied clinically without an irradiation device and can be locally injected into tumors in deep soft tissues [35]. Moreover, a previous report showed that PAM could provide a more homogeneous effect on affected cells than direct plasma irradiation [36].

According to previous studies, PAM showed an anti-tumor effect in vitro on various malignant tumor cells, including skeletal sarcomas [37]. Moreover, some in vivo studies using a tumor-bearing murine model have demonstrated that PAM effectively suppresses malignant tumors, including pancreatic cancer [38], ovarian cancer [39], osteosarcoma [40], and gastric cancer [41], without any side effects. Although the mechanism underlying the anti-tumor effect of PAM is not clear, cell apoptosis induced by intracellular reactive oxygen species (ROS) was suggested as one of the mechanisms [42].

However, whether PAM has an anti-tumor effect on synovial sarcoma and the underlying anti-tumor mechanism remain unknown. Considering problems with existing synovial sarcoma treatment options aforementioned, if PAM has an anti-tumor effect on synovial sarcoma, local PAM injection could be a valuable new option for synovial sarcoma treatment.

Therefore, this study aimed to investigate whether PAM has anti-tumor effects on synovial sarcoma and whether the anti-tumor mechanism involves cell apoptosis induced by intracellular ROS.

## 2. Materials and Methods

### 2.1. Cell Line and Cell Culture

The human synovial sarcoma cell line HS-SY-II [43], which had already completed cell line authentication, was purchased from RIKEN BioResource Research Center (Ibaraki, Japan) and used in this study. The cells were cultured in Dulbecco’s modified Eagle medium (DMEM; Thermo Fisher Scientific, Waltham, MA, USA) supplemented with 10% fetal bovine serum (FBS; Thermo Fisher Scientific) and 1% penicillin-streptomycin (Wako, Osaka, Japan) and maintained under 37 °C, 5% carbon dioxide, and humidified condition.

### 2.2. Plasma Irradiation and PAM Production

**Figure 1:**
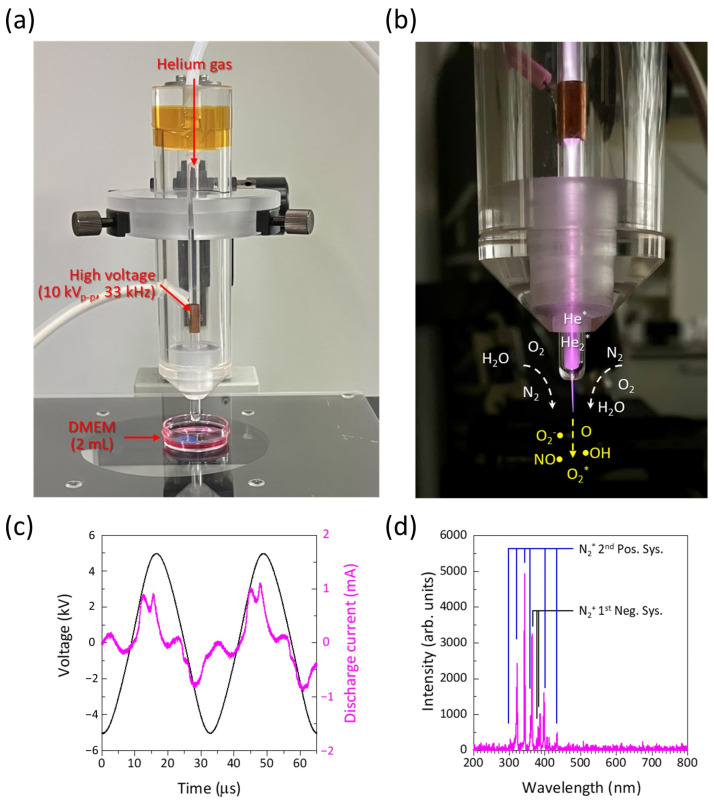
Non-thermal atmospheric pressure helium microplasma jet. (**a**) shows DMEM treated with non-thermal atmospheric pressure helium microplasma jet at fixed distance of 10 mm. (**b**) shows internal dielectric barrier helium gas discharge plasma and external plasma jet. Sufficient reactive oxygen and nitrogen species are generated in interaction between plasma and ambient air. (**c**) shows voltage and discharge current waveforms when helium microplasma jet was generated. (**d**) shows optical emission spectrum of external plasma jet, measured underneath nozzle exit. DMEM, Dulbecco’s modified Eagle medium.

A non-thermal atmospheric pressure helium microplasma jet was used to produce PAM, as shown in Figure 1a. Details of the plasma device are described in previous reports [20,21,22,44,45,46]. In brief, the microplasma jet consisted of a 150 mm long glass tube tapered from an inner diameter of 4 mm to 680 μm at the nozzle. The glass tube (borosilicate, Pyrex; As One Co., Osaka, Japan) has a 15 mm long metallic external ring electrode wound onto the glass tube at 50 mm from the end of the nozzle. The helium gas (99.98%, industrial grade) was fed into the glass tube. A capillary dielectric barrier discharge (DBD) microplasma shown in Figure 1b was generated when a sinusoidal high voltage of 10 kV_p-p_ (peak-to-peak) was applied to the external electrode at a fixed frequency of 33 kHz. The voltage and discharge current waveforms are shown in Figure 1c. From the voltage and current waveforms, the peak power of approximately 5 W was estimated. The typical optical emission spectrum, which shows mainly N_2_^*^ second positive system, N_2_^+^ first negative system, and a very weak emission peak of OH radical in Figure 1d, was measured.

For PAM production, 2 mL of DMEM (Wako) without FBS or antibiotics in a 35 mm dish was irradiated with plasma. The helium flow rate through the plasma jet nozzle and the distance between the nozzle tip and the medium surface were fixed at 0.58 L/min and 10 mm, respectively. For in vitro experiments, DMEM irradiated with plasma for 1, 3, and 5 min, i.e., PAM (1, 3, and 5 min), was used. For in vivo experiments, DMEM irradiated with plasma for 20 min, i.e., PAM (20 min), was used. In all the experiments, DMEM without plasma irradiation was used as the control for PAM.

### 2.3. Cell Viability Assay

The human synovial sarcoma cells were seeded in a 96-well plate at a density of 5 × 10^4^ cells/well with 100 μL of DMEM and cultured for 24 h. The medium was replaced with 100 μL of each PAM (1, 3, and 5 min) or DMEM, and then the cells were cultured for another 24 h. After 24 h incubation, the media in all the wells were replaced with 100 μL of DMEM. Cell viability was measured using the TACS MTT Cell Proliferation Assay (R&D Systems, Inc., Bio-Techne, Minneapolis, MN, USA) according to the manufacturer’s instructions. Briefly, 10 μL of MTT reagent was added to each well. After 2 h incubation, the contents of all the wells were removed and 100 μL of detergent reagent was added and gently mixed. After another 2 h incubation at room temperature in the dark, the absorbance at the wavelength of 570 nm for each well was measured by a microplate reader (Varioskan LUX; Thermo Fisher Scientific).

### 2.4. Intracellular ROS Detection and Quantification

To detect the intracellular ROS accumulation, 5-6-chloromethyl-2′,7′-dichlorodihydrofluorescein diacetate, acetyl ester (CM-H_2_DCFDA; Invitrogen, Thermo Fisher Scientific), an intracellular ROS detection reagent, was used. The cells were seeded in a 12-well plate at a density of 5 × 10^4^ cells/well in 1 mL of DMEM and cultured for 24 h. After 24 h incubation, the media in all wells were replaced with 1 mL of CM-H_2_DCFDA solution (10 µM, dissolved in phosphate-buffered saline [PBS]), and the cells were incubated for an hour in a dark place. Then, the CM-H_2_DCFDA solution was removed from all the wells, and the cells were cultured in 1 mL of PAM (5 min) or DMEM for 2 h in the dark. After the media in all the wells were replaced with 1 mL of DMEM, the cells were cultured for another 2 h. Next, the cells were observed using a BZ-X810 fluorescence microscope (Keyence, Osaka, Japan). Five fields per well were randomly selected, and the intracellular fluorescence intensity per cell was measured using the BZ-X Analyzer software version 1.1.1.8 (Keyence).

### 2.5. N-Acetyl-L-Cysteine Treatment for Intracellular ROS Inhibition

N-acetyl-L-cysteine (NAC; Wako), an intracellular ROS scavenger, was used to inhibit intracellular ROS. The cells were seeded in a 96-well plate at a density of 5 × 10^4^ cells/well with 100 μL of DMEM and cultured for 22 h. Then, the wells were divided into two groups: NAC (+) and NAC (−) wells. For the NAC (+) wells, 10 μL of the NAC solution dissolved in PBS was added to each well (4 mM), and the cells were incubated for 2 h in a dark place. The medium was replaced with 100 μL of PAM (1, 3, and 5 min) or DMEM supplemented with 10 μL of NAC solution as mentioned earlier. After 24 h incubation in a dark place, the media in all the wells were replaced with 100 μL of DMEM. Eventually, cell viability was measured using the TACS MTT Cell Proliferation Assay (R&D Systems, Inc.). For the NAC (−) wells, as the control for NAC, the same volume of PBS was added to the wells instead of the NAC solution.

### 2.6. Flow Cytometry for Cell Apoptosis Assay

The cells were seeded in a 6-well plate at a density of 1 × 10^6^ cells/well in 2 mL of DMEM and cultured for 24 h. The medium was replaced with 2 mL of PAM (1, 3, and 5 min) or DMEM, and the cells were incubated for 24 h. The cells, including those in the supernatant, were harvested using trypsin and washed with cold PBS. For the cell apoptosis assay using flow cytometry, the BD Pharmingen FITC Annexin V Apoptosis Detection Kit I (Becton, Dickinson and Company, Franklin Lakes, NJ, USA) was used according to the manufacturer’s instructions. Briefly, the cells were stained with 5 μL each of FITC Annexin V and Propidium Iodide (PI) for 15 min in a dark place, and the apoptotic rate was measured using flow cytometry (BD LSRFortessa X-20; Becton, Dickinson, and Company). The apoptotic rate was defined as the sum of the early and late apoptotic rates and calculated as the FITC Annexin V-positive cell rate.

### 2.7. Animal Experiments

All animal experiments were approved by the Institutional Animal Care and Use Committee of Osaka Metropolitan University (approval number: 22057) and were conducted in accordance with the Osaka Metropolitan University Animal Experimentation Regulations. Four-week-old female BALB/c nu/nu mice were purchased from Japan SLC, Inc. (Shizuoka, Japan), and 3 × 10^6^ HS-SY-II cells in 200 μL of PBS were subcutaneously injected into their right flank, which was the previously described way to achieve synovial sarcoma murine models [47]. Once tumor formation was achieved, the longest and shortest diameters of the tumors were measured once a week using an electronic caliper (Mitutoyo Corporation, Kanagawa, Japan), and the tumor volume was calculated as (longest diameter) × (shortest diameter)^2^/2, as previously described [48]. Once the tumor volume exceeded 100 mm^3^, the mice were randomly divided into PAM and control groups. In the PAM group, 200 μL of PAM (20 min) was subcutaneously injected around the tumor every day for 4 weeks. In the control group, an equal volume of DMEM was injected in place of PAM. The body weights of the mice were measured once a week to assess the side effects induced by PAM. After the 4-week treatment, the mice were sacrificed on day 29, and the tumors were resected and weighed.

### 2.8. Statistical Analysis

All experiments were repeated at least three times, and the data are presented as mean ± standard deviation. The data were analyzed with EZR software version 1.68 (Saitama Medical Center, Jichi Medical University, Saitama, Japan) using the Mann–Whitney *U* test, Kruskal–Wallis test, followed by the Bonferroni post hoc test and a linear mixed effect model. Statistical significance was set at *p* < 0.05.

## 3. Results

### 3.1. The Effect of PAM on HS-SY-II Cell Viability

**Figure 2:**
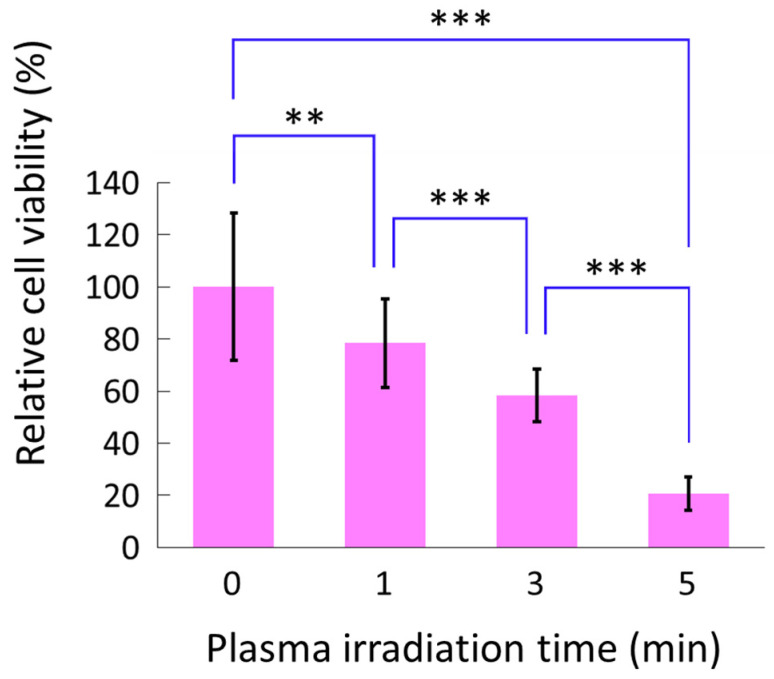
HS-SY-II cell viability after PAM or DMEM treatment. Cell viability after PAM treatment was normalized with that after DMEM treatment. ** *p* < 0.01, *** *p* < 0.001, Kruskal–Wallis test followed by Bonferroni post hoc test. PAM, plasma-activated medium; DMEM, Dulbecco’s modified Eagle medium.

Figure 2 shows the cell viability after PAM (1, 3, and 5 min) or DMEM treatment. Cell viability after PAM treatment significantly decreased in a plasma irradiation time-dependent manner compared with that after DMEM treatment. PAM (5 min) reduced cell viability to approximately 21% of that of the control (*p* < 0.001).

### 3.2. Intracellular ROS Accumulation Induced by PAM

**Figure 3:**
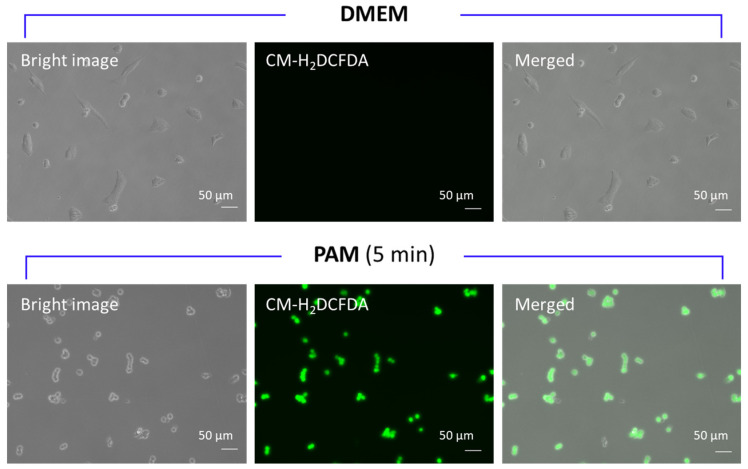
Representative fluorescence microscopic images of cells treated with PAM (5 min) or DMEM after CM-H_2_DCFDA pretreatment for observing intracellular ROS accumulation induced by PAM. Scale bars represent 50 μm. PAM, plasma-activated medium; DMEM, Dulbecco’s modified Eagle medium; CM-H_2_DCFDA, 5-6-chloromethyl-2’, 7’-dichlorodihydrofluorescein diacetate, acetyl ester; ROS, reactive oxygen species.

Figure 3 shows representative fluorescence microscopy images of cells treated with PAM (5 min) or DMEM after CM-H_2_DCFDA pretreatment. The intracellular fluorescence intensity after PAM treatment was 11,761.4 ± 1301.5-fold higher than that after DMEM treatment (*p* < 0.001), indicating that PAM induced significant intracellular ROS accumulation.

### 3.3. Involvement of Intracellular ROS Accumulation in Anti-Tumor Effect of PAM on HS-SY-II Cells

**Figure 4:**
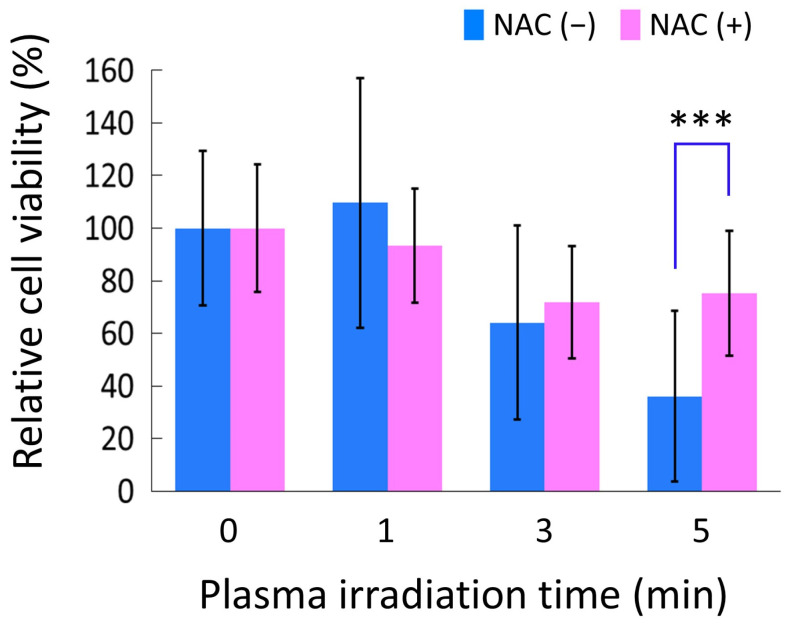
HS-SY-II cell viability after PAM or DMEM treatment with and without NAC, an intracellular ROS scavenger. The cell viability after each PAM treatment was normalized with that after the DMEM treatment. *** *p* < 0.001, Mann–Whitney *U* test vs. cell viability after each PAM treatment without NAC. PAM, plasma-activated medium; DMEM, Dulbecco’s modified Eagle medium; NAC, N-acetyl-L-cysteine; ROS, reactive oxygen species.

We examined whether intracellular ROS accumulation induced by PAM, as described in Figure 3, was involved in the mechanism underlying the anti-tumor effect of PAM on HS-SY-II cells, as described in Figure 2. Figure 4 shows the cell viability after PAM or DMEM treatment with and without NAC. The cell viability after PAM (5 min) treatment with NAC was approximately 39% higher than that without NAC (*p* < 0.001).

### 3.4. HS-SY-II Cell Apoptosis Induced by PAM

**Figure 5:**
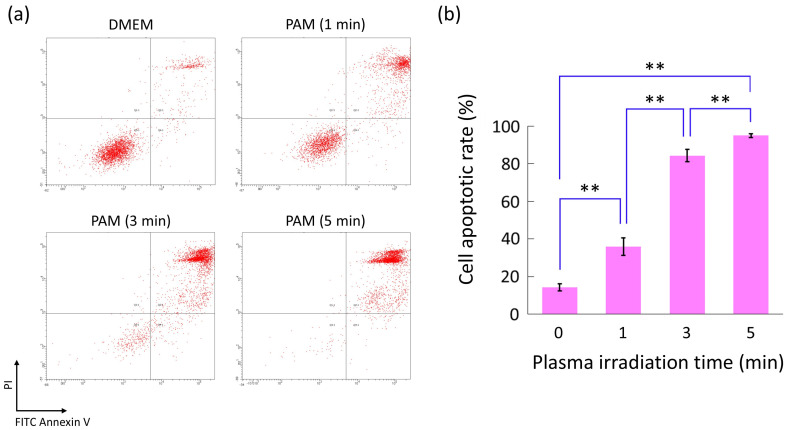
HS-SY-II cell apoptosis induced by PAM. (**a**) shows representative dot plots of cells stained with FITC Annexin V and PI after PAM or DMEM treatment. (**b**) shows cell apoptotic rate after PAM or DMEM treatment. ** *p* < 0.01, Kruskal–Wallis test followed by Bonferroni post hoc test. PAM, plasma-activated medium; PI, Propidium Iodide; DMEM, Dulbecco’s modified Eagle medium.

Figure 5a shows representative dot plots of the cells stained with Annexin V and PI after PAM or DMEM treatment, and Figure 5b shows the apoptotic rate of these cells. The cell apoptotic rate after PAM treatment significantly increased in a plasma irradiation time-dependent manner compared with that after DMEM treatment. In particular, PAM (5 min) significantly increased cell apoptosis compared with DMEM (*p* = 0.0025) and finally induced apoptosis in approximately 95% of the cells.

### 3.5. The Effect of PAM on Synovial Sarcoma Xenografts

**Figure 6:**
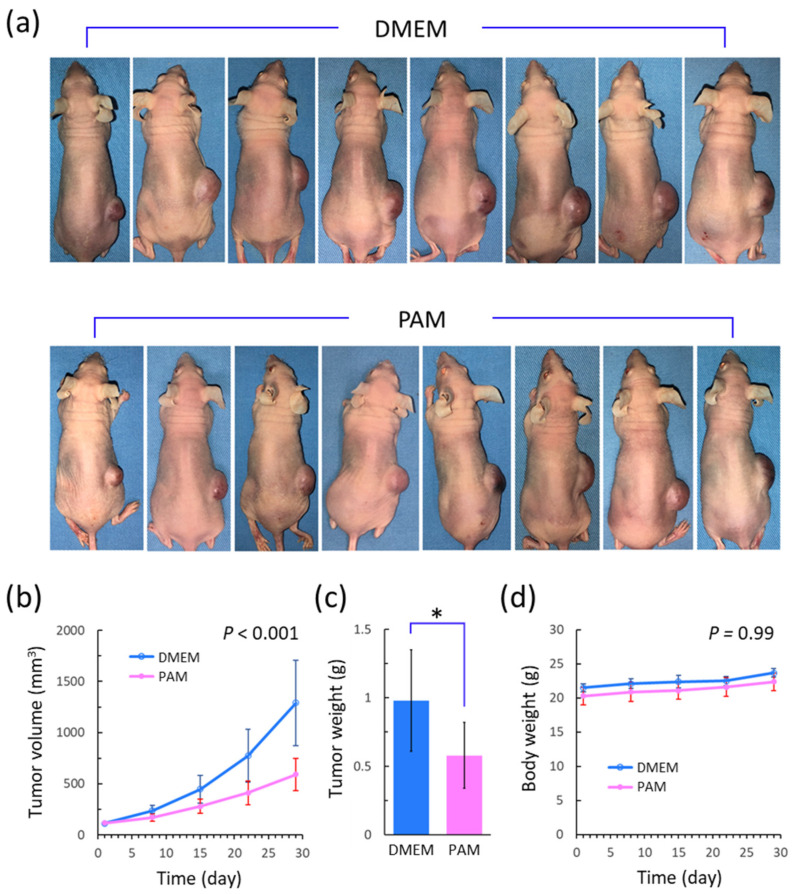
Effect of PAM on synovial sarcoma xenografts. (**a**) shows photographs of mice on day 29 in DMEM (upper) and PAM (lower) groups. (**b**) shows tumor volume through treatment period in two groups (linear mixed effect model). (**c**) shows resected tumor weight on day 29 in two groups. * *p* < 0.05, Mann–Whitney *U* test vs. control. (**d**) shows mouse body weight through treatment period in two groups (linear mixed effect model). PAM, plasma-activated medium; DMEM, Dulbecco’s modified Eagle medium.

Figure 6a shows the images of each mouse on day 29 in the DMEM and PAM groups, and Figure 6b shows the tumor volume throughout the treatment period. PAM significantly suppressed tumor growth compared with DMEM (*p* < 0.001). Finally, on day 29, PAM suppressed the tumor volume to approximately 46% of that in the DMEM group. Figure 6c shows the final tumor weights in both groups. PAM significantly reduced the final tumor weight to approximately 59% of that in the DMEM group (*p* = 0.031). Figure 6d shows the mouse body weights throughout the treatment period, which does not show a significant body weight loss in the PAM group (*p* = 0.99). In addition, mice in the PAM group did not show any visible side effects, such as diarrhea, anorexia, or abnormal action. In summary, PAM significantly suppressed synovial sarcoma in vivo without any apparent side effects.

## 4. Discussion

In this study, PAM treatment showed an anti-tumor effect on synovial sarcoma cells, which was partially inhibited by NAC, and induced intracellular ROS accumulation and cell apoptosis. Moreover, PAM significantly suppressed synovial sarcoma in xenograft murine models without any side effects.

Previous reports have demonstrated the anti-tumor effect of PAM in vitro on various malignant tumors, such as lung cancer [48], glioblastoma [49], gastric cancer [50], endometrial cancer [51], osteosarcoma, and Ewing’s sarcoma [37]. In addition to killing tumor cells, PAM reduces stem cell properties of malignant cells related to cancer metastasis and recurrence [52]. Moreover, previous reports have demonstrated the anti-tumor effect of PAM in vivo. Similarly to our study, some studies have demonstrated that local PAM injection significantly suppresses cancer, including breast cancer [36], pancreatic cancer [38], ovarian cancer [39], lung cancer [48], and melanoma [53], in xenograft murine models. In addition to suppressing the tumor itself, intraperitoneal injection of PAM decreases the peritoneal metastasis of cancers by reducing cell migration and adhesion ability [41,54]. In this study, we showed a significant anti-tumor effect of PAM on synovial sarcoma in vitro and in vivo, which supports findings of previous reports. This is the first report to assess the anti-tumor effect of PAM on synovial sarcoma.

Previous studies using various malignant cells demonstrated that plasma generated ROS inside the cells and caused cell apoptosis through various mechanisms, including DNA fragmentation, mitochondrial injury, and cell cycle arrest [25,38,42,55,56,57]. Similarly to previous reports, our results also suggest that intracellular ROS accumulation induced by PAM causes cell apoptosis, which is one of the anti-tumor mechanisms of PAM in synovial sarcoma cells. However, treatment with NAC did not completely inhibit cell death. This result indicates that some anti-tumor mechanisms do not involve intracellular ROS, which is considered to be primarily responsible for the anti-tumor mechanism of PAM [39,48,50,58]. Given that PAM generates not only ROS but also reactive nitrogen species (RNS) inside cells [32,59], RNS and other intracellular components may also have been involved in the anti-tumor mechanisms in our study. In addition to intracellular ROS production, several previous reports have suggested various other anti-tumor mechanisms of plasma-activated solutions. One of these reports suggested that the anti-tumor mechanism of PAM was related to the newly generated substance produced by plasma treatment of tryptophan and methionine, which are the components of cell culture medium [60]. Another report indicated that plasma-activated Ringer’s lactate solution caused cell death without relying on oxidative stress in glioblastoma cells and directly downregulated the phosphoinositide 3-kinase (PI3K)-AKT signaling pathway, intracellular survival, and proliferation pathways [58]. Other reports have demonstrated that the plasma-activated solution first stimulates proteins on cell membranes, including receptors and transporters, and then these affected proteins alter intracellular signaling pathways, including the PI3K-AKT and RAS-MAPK signaling pathways, consequently altering intracellular metabolism and gene expression related to the cell cycle or cell survival [32,59,61]. In summary, the mechanisms underlying the anti-tumor effect of PAM are not completely understood, and further research is needed to investigate anti-tumor mechanisms other than intracellular ROS.

Recently, treatments for synovial sarcoma have continued to be developed. The primary treatment options for synovial sarcomas are surgical resection, chemotherapy, and radiotherapy. Of these three options, surgical resection with negative margins remains the most effective treatment for synovial sarcoma [3]. However, it can often be challenging because synovial sarcoma tends to occur in the deep soft tissues of the extremities [1]. Moreover, surgery can be invasive and difficult to perform in patients with a poor general condition. The second and third options, chemotherapy and radiotherapy, were reported to be partially effective for synovial sarcoma treatment [3,4,5]; however, they were reported to have many serious side effects [7,8,9]. For example, while doxorubicin and ifosfamide, the first-line chemotherapy agents for synovial sarcoma, are moderately effective, they are cytotoxic and cause various clinical side effects, including nausea, fatigue, anemia, leukopenia, renal dysfunction, and impaired fertility [3,5,8,9]. Moreover, it is controversial whether radiotherapy is effective in improving the prognosis of patients with synovial sarcoma [4,6], and irradiated tissue often sustains adverse effects, including fibrosis, edema, and joint stiffness [7]. Taken together, the existing treatment options still have various problems.

Here, we demonstrated the anti-tumor effects of PAM on synovial sarcoma without any side effects. In support of our results, some previous studies demonstrated that plasma showed a “selective” anti-tumor effect on malignant cells and did not suppress healthy cells [24,29,36,62]. These studies suggested that one reason for such selectivity is that intracellular ROS levels are higher in malignant cells than in healthy cells and that malignant cells are more easily affected by intracellular ROS induced by plasma [36,63]. Another possible reason is that some aquaporins, channels on cell membranes that transport hydrogen peroxide into cells, are overexpressed in malignant cells, and these overexpressed aquaporins facilitate ROS accumulation in malignant cells compared with healthy cells [36,64,65]. Our results and such previous literature suggested the possibility of “plasma medicine,” including PAM, becoming the fourth option for synovial sarcoma treatment. Only one previous in vitro study showed that direct plasma irradiation of synovial sarcoma cells was effective [66]. However, direct plasma irradiation can be difficult to apply clinically to synovial sarcoma because of its common location in deep soft tissues [1]. Therefore, PAM, rather than direct plasma irradiation, has the potential to be an effective treatment option for patients with synovial sarcoma.

Although PAM significantly suppressed synovial sarcoma in vivo without side effects, PAM alone did not completely inhibit tumor growth or eliminate tumors. Surgery and chemotherapy could be unavailable for patients with poor physical conditions, and PAM could be used as a salvage treatment option for such patients. Moreover, wide resection with sufficient margins for synovial sarcoma is often difficult because of its common deep location in the body [1]. A previous clinical study showed that direct plasma irradiation of the surgical margin after malignant solid tumor resection effectively suppresses local tumor recurrence without adverse effects [67]. Considering that PAM can be easier to apply clinically than direct plasma irradiation because it is a liquid and can be sprayed on surgical margins without an irradiation device, the combination of surgery and postoperative PAM treatment was suggested to be promising. In addition, as already mentioned, existing chemotherapy drugs are cytotoxic and frequently cause severe side effects [3,5,8,9]. Therefore, even though PAM alone is not sufficient to treat synovial sarcoma, the combination of PAM and chemotherapy may reduce the dose of cytotoxic chemotherapy drugs and consequently reduce side effects. In fact, some previous studies have attempted combination treatment with specific chemotherapy drugs and PAM for some types of tumors. One study demonstrated that PAM significantly enhances the anti-tumor effects of sorafenib, doxorubicin, and cisplatin for hepatocellular carcinoma cells. In particular, the combination treatment with cisplatin and PAM showed an additive anti-tumor effect. As a reason for this additive effect, they suggested that PAM could inhibit the repair of DNA damage induced by alkylating drugs, including cisplatin [68]. Another in vivo study reported that the combination treatment with cisplatin and PAM significantly suppressed ovarian cancer compared with cisplatin or PAM alone, without adverse side effects [52]. Another study demonstrated that PAM not only enhanced the anti-tumor effect of doxorubicin on breast cancer but also decreased the hepatotoxicity and renal toxicity induced by doxorubicin [69]. Further research is needed to assess the effectiveness of the combination treatment with chemotherapy and PAM for synovial sarcoma. Moreover, radiotherapy frequently causes various side effects [7], and combination treatment with PAM and radiotherapy may reduce the irradiation area and consequently decrease side effects. Taken together, PAM is suggested to be a new promising option for synovial sarcoma treatment. However, further investigation is necessary for its clinical application, including combination treatment with PAM and existing treatments.

This is the first study to investigate the anti-tumor effect of PAM on synovial sarcoma, and both in vivo and in vitro experiments were needed to explore the anti-tumor mechanisms. We wanted to use the same plasma-activated solution for the in vitro and in vivo experiments because the anti-tumor efficacy of plasma-activated solutions varies depending on the solution irradiated with plasma [32]. Therefore, plasma-irradiated “DMEM,” i.e., PAM, was used for in vivo experiments as for in vitro experiments. However, cell culture media are not intended for human use. Previous studies have performed in vivo experiments using solutions other than DMEM. One study showed that plasma-activated Soldem 3A significantly reduced the growth of murine osteosarcoma [70]. Another study showed that plasma-activated Ringer’s lactate solution significantly suppressed cervical cancer [71]. For synovial sarcoma, we plan to perform additional clinical in vivo studies using a solution clinically approved for human use.

This study has a few limitations. First, we prepared tumors subcutaneously for the in vivo experiments, regardless of the fact that synovial sarcoma often occurs in deep soft tissues [1]. This may have provided the tumors with a surrounding environment that was different from that of an actual synovial sarcoma, such as blood flow around the tumors. However, subcutaneous tumor formation allowed us to measure tumor size easily without using computed tomography with radiation exposure, which may have affected our results. Second, immunodeficient mice were used for the in vivo experiments. Some studies have shown that plasma enhances the anti-tumor immune system [72,73,74], but we could not consider this anti-tumor immune effect in this study because the immune system was suppressed in our mice. If we had used immunocompetent mice in the in vivo experiments, PAM could have suppressed synovial sarcoma more strongly. Yet, using human cells instead of murine cells was considered to make this study more clinically valuable. Third, our in vivo assessment of the side effects was insufficient. However, in our in vivo experiments, we used a plasma-irradiated “cell culture medium” to ensure consistent anti-tumor efficacy, and it was not approved for human use. In the future, we will conduct similar in vivo experiments using a different solution that can be clinically used in humans instead of DMEM and assess other side effects, including pathological changes in organs and abnormalities in blood test results.

## 5. Conclusions

PAM showed anti-tumor effects on synovial sarcoma, both in vitro and in vivo, with no side effects. In addition, the anti-tumor mechanism of PAM was suggested to involve cell apoptosis mainly due to intracellular ROS accumulation. These results indicate that PAM could be a valuable new option for the effective treatment of synovial sarcoma.

## Data Availability

The original contributions presented in the study are included in the article, further inquiries can be directed to the corresponding author.
